# Subcellular Sequestration and Impact of Heavy Metals on the Ultrastructure and Physiology of the Multicellular Freshwater Alga *Desmidium swartzii*

**DOI:** 10.3390/ijms160510389

**Published:** 2015-05-07

**Authors:** Ancuela Andosch, Margit Höftberger, Cornelius Lütz, Ursula Lütz-Meindl

**Affiliations:** 1 Plant Physiology Division, Cell Biology Department, University of Salzburg, Hellbrunnerstrasse 34, 5020 Salzburg, Austria; E-Mails: ancuela.andosch@sbg.ac.at (A.A.); margit.hoeftberger@sbg.ac.at (M.H.); 2 Institute of Botany, Faculty of Biology, University of Innsbruck, Sternwartestrasse 15, 6020 Innsbruck, Austria; E-Mail: cornelius.luetz@uibk.ac.at

**Keywords:** aluminum, cadmium, chromium, copper, *Desmidium swartzii*, electron energy loss spectroscopy, TEM, zinc

## Abstract

Due to modern life with increasing traffic, industrial production and agricultural practices, high amounts of heavy metals enter ecosystems and pollute soil and water. As a result, metals can be accumulated in plants and particularly in algae inhabiting peat bogs of low pH and high air humidity. In the present study, we investigated the impact and intracellular targets of aluminum, copper, cadmium, chromium VI and zinc on the filamentous green alga *Desmidium swartzii*, which is an important biomass producer in acid peat bogs. By means of transmission electron microscopy (TEM) and electron energy loss spectroscopy (EELS) it is shown that all metals examined are taken up into *Desmidium* readily, where they are sequestered in cell walls and/or intracellular compartments. They cause effects on cell ultrastructure to different degrees and additionally disturb photosynthetic activity and biomass production. Our study shows a clear correlation between toxicity of a metal and the ability of the algae to compartmentalize it intracellularly. Cadmium and chromium, which are not compartmentalized, exert the most toxic effects. In addition, this study shows that the filamentous alga *Desmidium* reacts more sensitively to aluminum and zinc when compared to its unicellular relative *Micrasterias*, indicating a severe threat to the ecosystem.

## 1. Introduction

Fresh water algae inhabiting acid peat bog ponds are extremely endangered by entry of metallic pollutants arising from increasing traffic, industrial production or agricultural practices. Both, aerosols, which are released particularly in areas of high humidity [1], and disposal of waste water [2] are the main sources for metal contamination of moors and wetlands. Metals such as cadmium, copper, chromium, aluminum and zinc have been shown to evoke severe damage to algal metabolism by displacing essential ions, interacting with enzymes, hampering uptake of essential nutrients or generally influencing intracellular ionic balance (see [3,4,5,6] and references herein). Formation of reactive oxygen species (ROS), inhibition or retardation of growth and cell division, impairment of photosynthetic activity and cell death may be the consequences of metal impact [7,8,9,10,11]. The magnitude of the damage depends on how far the metal is taken up into the cell, how it is distributed, what it targets and whether the alga is capable of compartmentalizing or physiologically detoxifying a metal pollutant. Investigation on intracellular localization of metals at high resolution as provided by analytical TEM is therefore required for understanding the danger of metal impact, in order to obtain insight into metal detoxification and to find solutions for amelioration of metal effects (see e.g., [12]).

Recent studies on the unicellular desmid *Micrasterias*, which has served as a cell biological model system since many years, have revealed a variety of cellular responses to heavy metal impact. Sulfate solutions of the metals Zn, Al, Cr, Cu and Cd evoke adverse effects on cell growth, cytomorphogenesis, cell division rate and photosynthetic activity in a dose dependent manner [3,4,5,13]. By means of TEM-coupled electron energy loss spectroscopy (EELS) it was shown that upon experimental Zn exposure for three weeks, the metal is found in the cell wall, in the vacuole and in mucilage vesicles indicating that Zn is taken up into the cell quite readily. *Micrasterias* is obviously capable of detoxifying Zn to a limited degree by storage in cell walls and vacuoles and by excretion via mucilage vesicles with an upper limit at 30 µM [13]. Al was only found in primary and secondary cell walls after three weeks indicating a filter capacity of the apoplast allowing at least some cell divisions at a concentration of 20 µM. The tolerance of *Micrasterias* for Cu was particularly limited, as concentrations higher than 0.3 µM for three weeks already led to cell death. Below this concentration, however, the cells were able to grow quite normally and both cell division rates and photosynthetic activity were only slightly reduced [13]. Among the metals investigated for their distribution in *Micrasterias*, Cu was compartmentalized the best. It was measured in cell wall pores, starch grains and mucilage vesicles. Cr effects on *Micrasterias* depended on the oxidation state in which the metal was applied. Both CrIII and CrVI inhibited cell growth but only CrVI impaired cell division. CrVI was detected in electron dense precipitates located in bag-like structures along the inner side of the cell wall together with Fe and elevated levels of oxygen after a three weeks exposure of the cells to CrVI [4]. Together with measurements by atomic emission spectroscopy this result indicates that Cr is accumulated and sometimes also extruded from *Micrasterias* cells in the form of a chromium-iron compound. As the Cr:Fe ratio is shifted in favor of Cr during Cr exposure, its uptake via Fe-transporter into *Micrasteria*s is implied. Additionally, pre-exposure of the cells to Fe decreased the Cr uptake and intracellular Cr accumulations were no longer detectable by analytical TEM [5]. Cd, which exerted the most pronounced effects on cell growth, cell division and photosynthesis of *Micrasterias*, in a nanomolar range during a three-week exposure, could not be detected in any intracellular compartment [3,13]. This leads to the conclusion that Cd is evenly distributed in *Micrasterias* in a concentration range not measureable by the highly sensitive methods employed. In this way the metal may affect a large number of different metabolic pathways, which explains its extreme toxicity in *Micrasterias*. It has further been shown that Cd leads to a complete and specific disintegration of dictyosomes from *cis* to *trans* and to the induction of autophagy. However, these adverse Cd effects on ultrastructure and its impairment of photosynthetic activity can be ameliorated by pre-treatment of *Micrasterias* cells with Ca [3]. Also dictyosomal disintegration can be mimicked by thapsigargin, an inhibitor of Ca^2+^-ATPase, suggesting that the adverse Cd effects are based on a disturbed Ca homeostasis by displacement of Ca by Cd. As Fe ameliorates adverse Cd effects as well in *Micrasterias,* it was concluded that Cd is taken up into cells via both Ca and Fe transporters [5]. The only Cd detoxification mechanism that has been detected in *Micrasterias* so far is an increase in the phytochelatins PC_2_, PC_3_ and PC_4_ in Cd stressed cells [14].

While the results obtained in *Micrasterias* provide important information on cellular and subcellular reactions of a well-investigated model organism to heavy metal impact, the contribution of unicellular algae such as *Micrasterias* to biomass production in a peat bog is relatively low. The few multicellular filamentous desmids inhabiting the same ecosystems as *Micrasterias* are much more effective in biomass production and additionally represent an evolutionary link to other groups of multicellular green algae and further crossover to higher plants [15]. Among those, *Desmidium* *swartzii*, frequently associated with desmids like *Micrasterias* in natural habitats, seems particularly interesting to study heavy metal influence. Comparison of the results with those obtained in *Micrasterias* will yield a more general view on cellular heavy metal impact and on the developing threat to the ecosystem. Early investigations [16] indicated that filamentous desmids like *Desmidium* are less resistant against heavy metals than the unicellular members of this family. If this result proves to be true the threat to the ecosystem is increased as it may rapidly break down when a large algae population is irretrievably demolished.

*Desmidium* consists of a long, spirally twisted filament of small indented cells (length approx. 20 µm) with cell wall properties of a typical *Desmidiaceae*. In the present study we investigated the influence of Zn, Al, Cu, Cd and Cr on biomass production, morphogenesis, ultrastructure and physiology of *Desmidium.* Sequestration of metals in cell compartments is analyzed by TEM-coupled EELS and electron spectroscopic imaging (ESI) techniques as well as by morin hydrate fluorescence by confocal laser scanning microscopy (CLSM) in the case of Al and Zn. Metal distribution and sequestration patterns are compared to the results obtained in *Micrasterias.* In this way we aim to obtain a comparative view on metal toxicity between unicellular and multicellular bog algae. As a basis for our study, morphogenesis and cell wall formation of *Desmidium* is described herein for the first time.

## 2. Results

### 2.1. Cell Development and Cell Wall Composition of Untreated Desmidium Filaments

**Figure 1:**
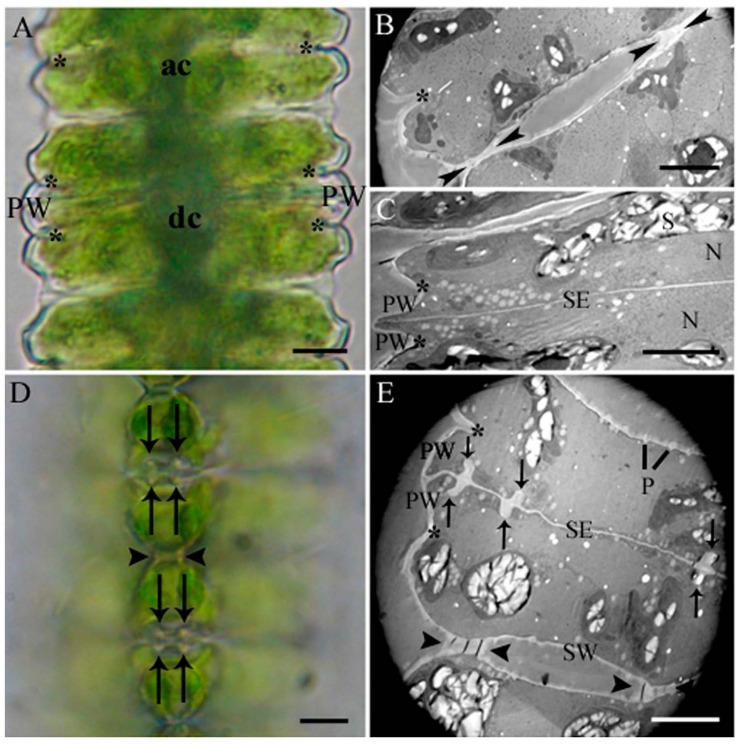

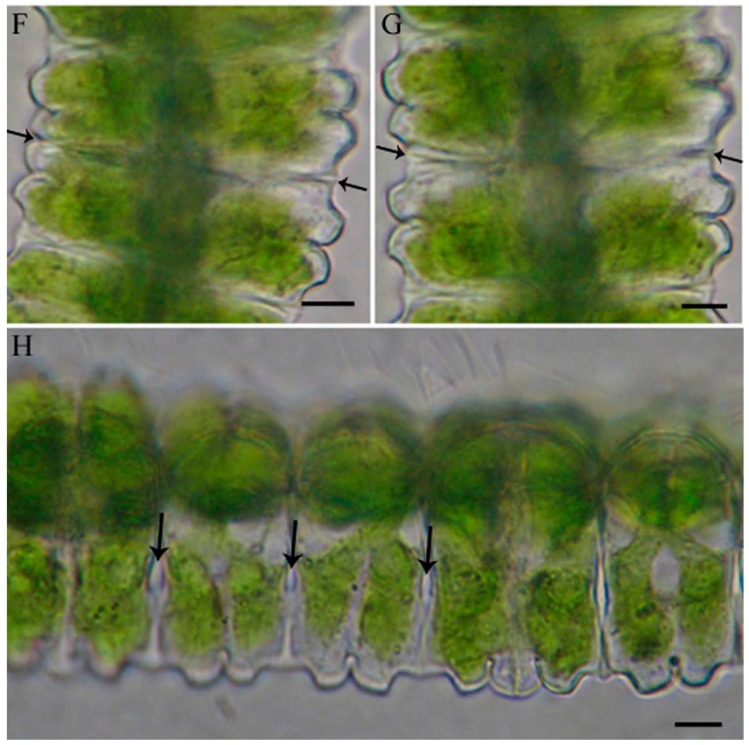
Details of cell development of *Desmidium* in light and electron microscopy. Light microscopic images show the same stages of development as electron micrographs aside. (**A**) Part of a *Desmidium* filament with an adult (ac) and a dividing cell (dc). The cell shape is established by a newly-formed primary wall in the isthmus (asterisks); (**B**) Two adult cells (cross section) connected by primary wall remnants (arrowheads). Isthmus marked by asterisk; (**C**) Newly-formed primary wall emanating from the isthmus (asterisks) and young septum; (**D**) Polar view of wall cylinder (arrows) in two developing cells and connecting cell wall (arrowheads); (**E**) Ultrastructure of dividing *Desmidium* cells (cross section). The newly-formed primary wall arising from the isthmus (asterisks), and outer and inner cell wall cylinders (arrows) perpendicular to the septum are visible. Remaining cell wall connection between two cells indicated by arrowheads; (**F**) Splitting of the connecting cell wall visible at the cell edges. On the left side the wall has split only slightly, on the right side more distinctly (arrows); (**G**) also, the left side has split completely. The arrows now point at the remaining connecting wall; (**H**) The cell wall within the inner wall cylinder has split as well, recognizable by the appearing background (arrows). N: Nucleus; P: Pores; PW: Primary wall; S: Starch grains; SE: Septum; SW: Secondary wall. Bar = 5 µm.

**Figure 2:**
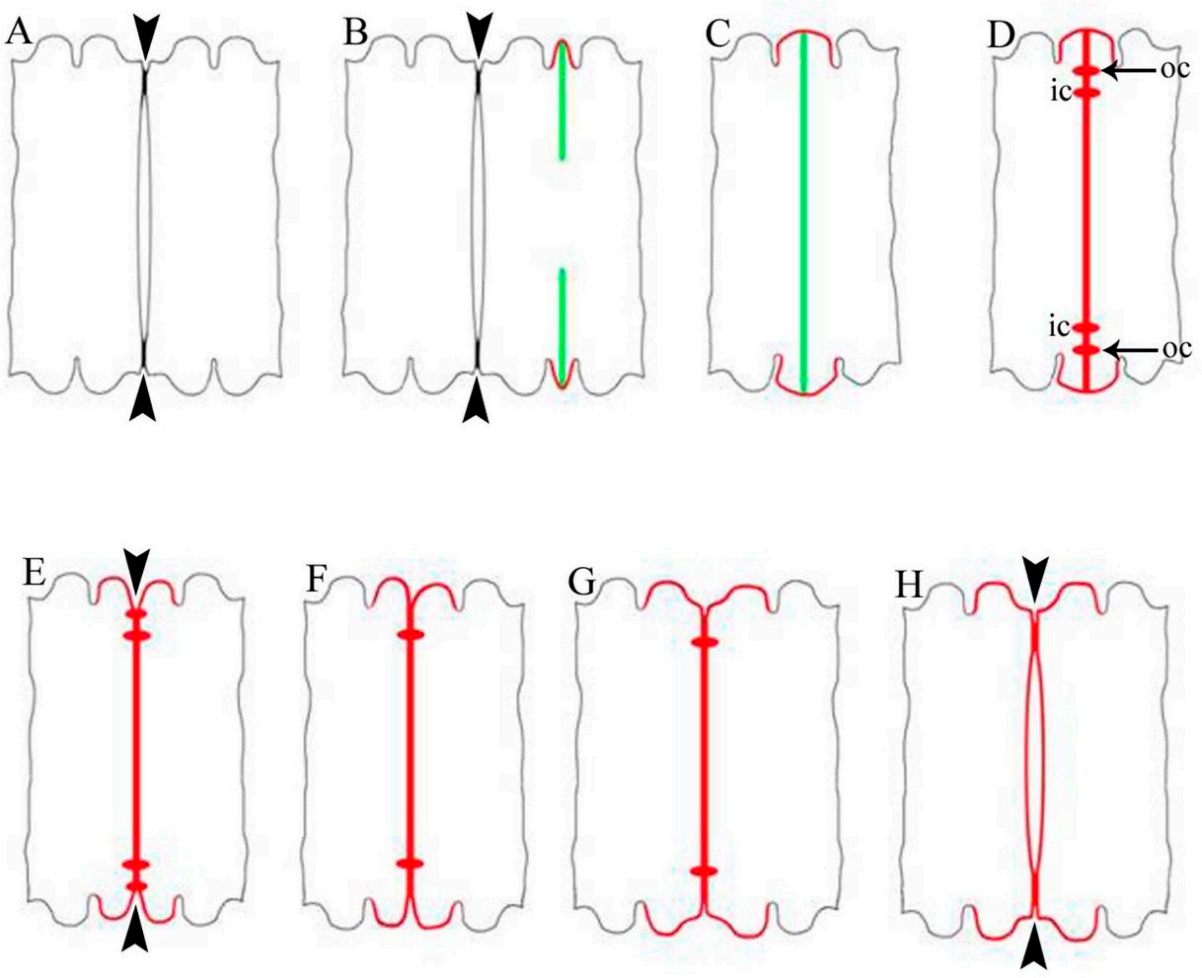
Schematic reconstruction of cytomorphogenesis in *Desmidium* (cross section). (**A**) Two adult cells connected by primary wall remnants (arrowheads); (**B**–**D**) Morphogenesis by new wall formation; (**B**) Developing primary wall (red) in the isthmus and outgrowing septum (green); (**C**) The cell halves are fully separated by a septum; (**D**) Outer (oc) and inner (ic) cell wall cylinder are developed perpendicular to the septum; (**E**–**H**) Morphogenesis by modeling of the newly-formed wall; (**E**) Connecting cell wall has split (arrowhead); (**F**) The outer cylinder has fully split and unfolded; (**G**) The final cell shape is formed at the cell edges; and (**H**) The remaining connecting wall is split and the inner cylinder is unfolded, only the wall (arrowheads) lying between inner and outer cylinder remains, cytomorphogenesis is completed.

**Figure 3:**
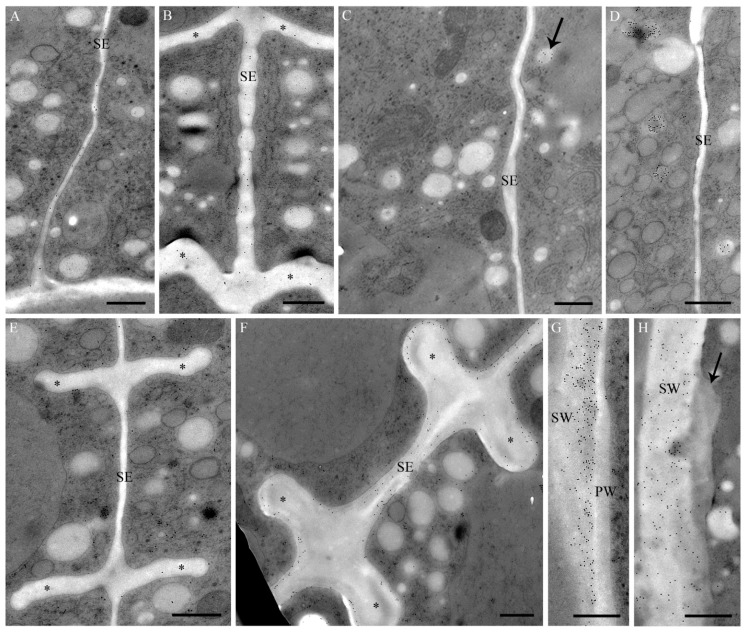
(**A**–**D**) Immunogold labeling with different JIM antibodies during cell wall formation in *Desmidium*; and (**E**–**H**) immunogold labeling with BG1 (cross sections). (**A**) At an early stage the thin septum is labeled by JIM 5; (**B**) In a subsequent stage septum and newly-formed cell wall cylinders (asterisks) are labeled by JIM 7; (**C**) A particular population of vesicles in the area of the septum is slightly stained by JIM 8 (arrow). No staining of the septum by JIM 8; (**D**) Particular vesicle population stained by JIM 13. No staining of the septum by JIM 13; (**E**) Septum and cell wall cylinders (asterisks) in an early developmental stage. No labeling of the primary wall by BG1; (**F**) Septum and cell wall cylinders (asterisks) in a later developmental stage. The secondary wall is clearly labeled by BG1, the central part, consisting of the primary wall, remains unstained; (**G**) Newly-formed primary wall in the isthmus with adjacent secondary wall. The secondary wall is strongly labeled by BG1, the primary wall remains unstained; (**H**) Secondary wall of a ZnSO_4_-treated cell with abnormal depositions (arrow). The secondary wall is labeled by BG1, the abnormal deposition not. PW: Primary wall; SE: Septum; SW: Secondary wall. Bar = 0.5 µm.

Cell development in *Desmidium* starts with a first initiating primary wall formation in the isthmus area, which subsequently leads to the basic shape of the new semicell (Figure 1A–C and Figure 2A,B). Emanating from this newly-built primary wall a septum is formed, which separates the two mirror-imaged cell halves (Figure 1C and Figure 2B,C). In a particular distance to the septum rim, two cell wall cylinders (Figure 1D,E and Figure 2D) develop perpendicular to the septum. The septum as well as the cell wall cylinders are underlaid by a secondary wall (Figure 3F). As demonstrated in the schematic drawing in Figure 2E–H, the final cell shape results from a splitting of the cell wall, an unfolding of the cylinder and a rounding at the edges. The splitting of the wall starts at its outer margin and continues to the outer cell wall cylinder. Here it changes direction and tears apart the cell wall of the cylinder perpendicular to the original septum. The outer cylinder unfolds and thus becomes part of the outer cell wall together with the former septum (Figure 1F,G). The inner cell wall cylinder as well as the wall lying in between also split and the cylinder unfolds, with the result that finally the two newly-formed cells are connected only by the cell wall that lies between inner and outer cylinder (Figure 1H and Figure 2H).

Details of the cell wall composition in *Desmidium* are analyzed using antibodies against various cell wall components. Both JIM 5 (specific for low methyl-esterified pectins) and JIM 7 (specific for highly methyl-esterified pectins) label the growing primary cell wall.

JIM5 labeling was shown in a young septum wall (Figure 3A), JIM 7 labeling in the cell wall cylinders and in the septum (Figure 3B). JIM 13 and JIM 8 specific for arabinogalactans, label a particular population of secretory vesicles (Figure 3C,D). BG1 recognizing (1-3,1-4)-ß-d-glucans (hemicelluloses) reveals a distinct staining of the secondary wall, whereas the primary wall is not labeled (Figure 3E–G).

### 2.2. Heavy Metal Effects on Biomass Production

**Figure 4:**
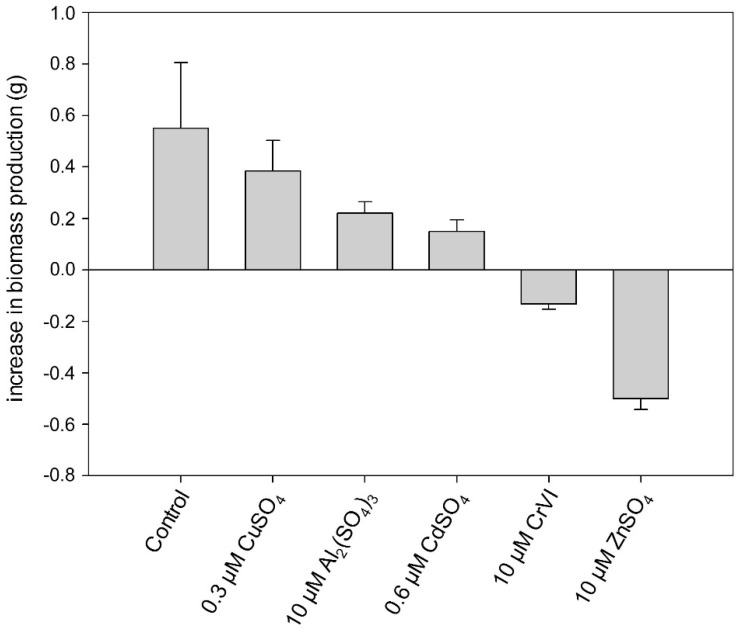
Increase in biomass production during exposure of *Desmidium* filaments to different metals in liquid culture medium for 21 days. Data are the means of three independent experiments ± standard error (SE).

The biomass of untreated *Desmidium* filaments reveals an increase of 0.55 g/100 mL Erlenmeyer flask (averaged from three independent experiments) within 21 days when the algae are grown in liquid culture medium, under standard conditions (see Experimental Section; Figure 4, Table 1). However, during the same period when *Desmidium* was treated with 0.3 µM CuSO_4_, the biomass increase was reduced to 0.39 g, when exposed to 10 µM Al_2_(SO_4_)_3_ the biomass increased only 0.22 g and after incubation in 0.6 µM CdSO_4_ only 0.15 g. A decrease of the initial biomass took place during exposure of the algae filaments to Cr and Zn indicating lethal effects. Treatment with 10 µM K_2_Cr_2_O_7_ (CrVI) decreased the initial biomass by 0.13 g and 10 µM ZnSO_4_ by 0.5 g (Figure 4, Table 1).

### 2.3. Heavy Metal Effects on Photosynthetic Electron Transport Efficiency (PSII)

**Figure 5:**
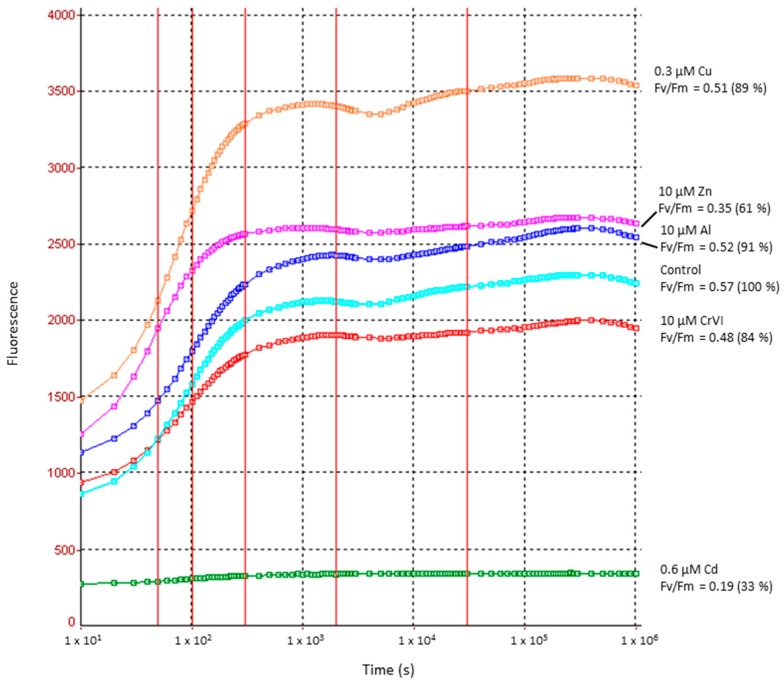
Photosynthetic electron transport efficiency in PSII in controls and heavy metal treated *Desmidium* filaments after 21 days measured by fast chlorophyll fluorescence.

As shown in Figure 5, the efficiency of PSII measured by *F*_v_/*F*_m_ ratio dropped only slightly in case of Al and Cu when compared to untreated controls (see also Table 1). Whereas the control *F*_v_/*F*_m_ value in *Desmidium* is 0.57 (=100%), it is reduced to 0.52 (91% of the control value) during 21 days exposure to Al and to 0.51 (89%) after treatment with Cu. CrVI caused a reduction of PSII electron transport efficiency to 0.48 (84%) and Zn to 0.35 (61%). A reduction of the *F*_v_/*F*_m_ value to 0.19 (33% of the control) by Cd indicates a severe impairment of photosynthetic activity by this metal.

### 2.4. Changes in Ultrastructure and Organelle Morphology after Heavy Metal Exposure

Only Zn, Al and CrVI induced light microscopically visible effects in terms of chloroplast contractions and occasional slight cell shape malformations in *Desmidium* (data not shown). After all other treatments, *Desmidium* filaments did not differ from controls when investigated in the light microscope.

**Figure 6:**
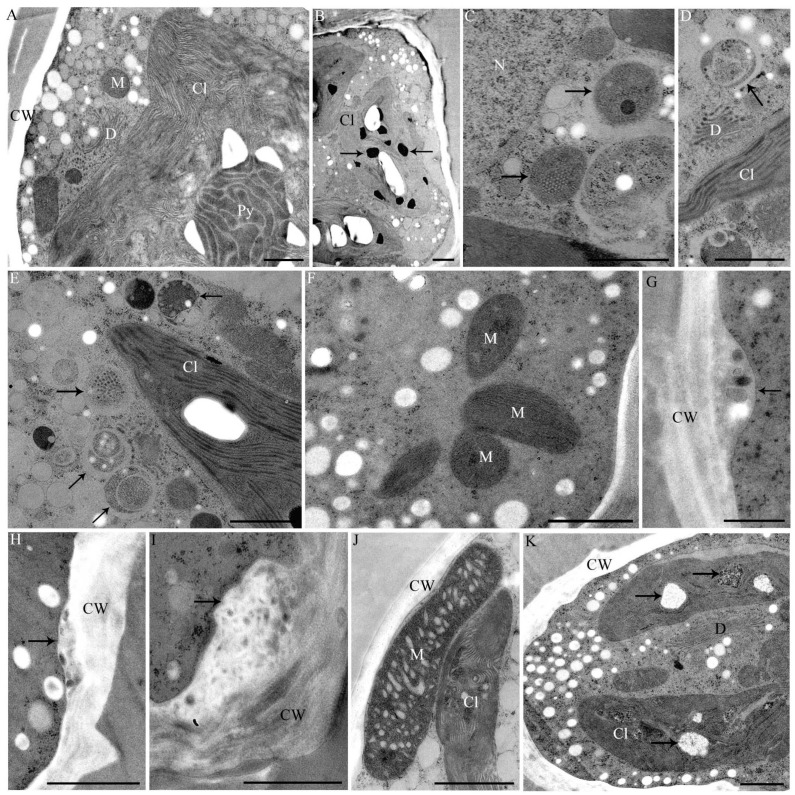
TEM micrographs of *Desmidium.* (**A**) Untreated control; (**B**) Treated with 10 µM Al_2_(SO_4_)_3_ for 21 days; (**C**–**E**) 0.6 µM CdSO_4_ for 21 days; (**F**–**G**) 10 µM CrVI for 35 days; (**H**,**K**) 0.3 µM CuSO_4_ for 21 days; and (**I**,**J**) 10 µM ZnSO_4_ for 21 days; (**B**) Chloroplast with abnormal dark inclusions (arrows); (**C**) Altered mitochondria (arrows) and (**D**,**E**) autophagosomal-like structures (arrows); (**F**) Cr-treated cell without visibly affected mitochondria; (**G**–**I**) Abnormal depositions on the cytoplasmic site of the cell wall (arrows) induced by Cr (**G**), Cu (**H**) and Zn (**I**); (**J**) Enlarged mitochondrion after Zn treatment, close to the chloroplast. Cristae are distinctly enlarged and bloated; (**K**) Vacuole-like structures in the chloroplast (arrows). Cl: Chloroplast; CW: Cell wall; D: Dictyosome; M: Mitochondrion; N: Nucleus; Py: Pyrenoid. Bar = 1 µm, except (**D**,**E**) = 0.5 µm.

**Figure 7:**
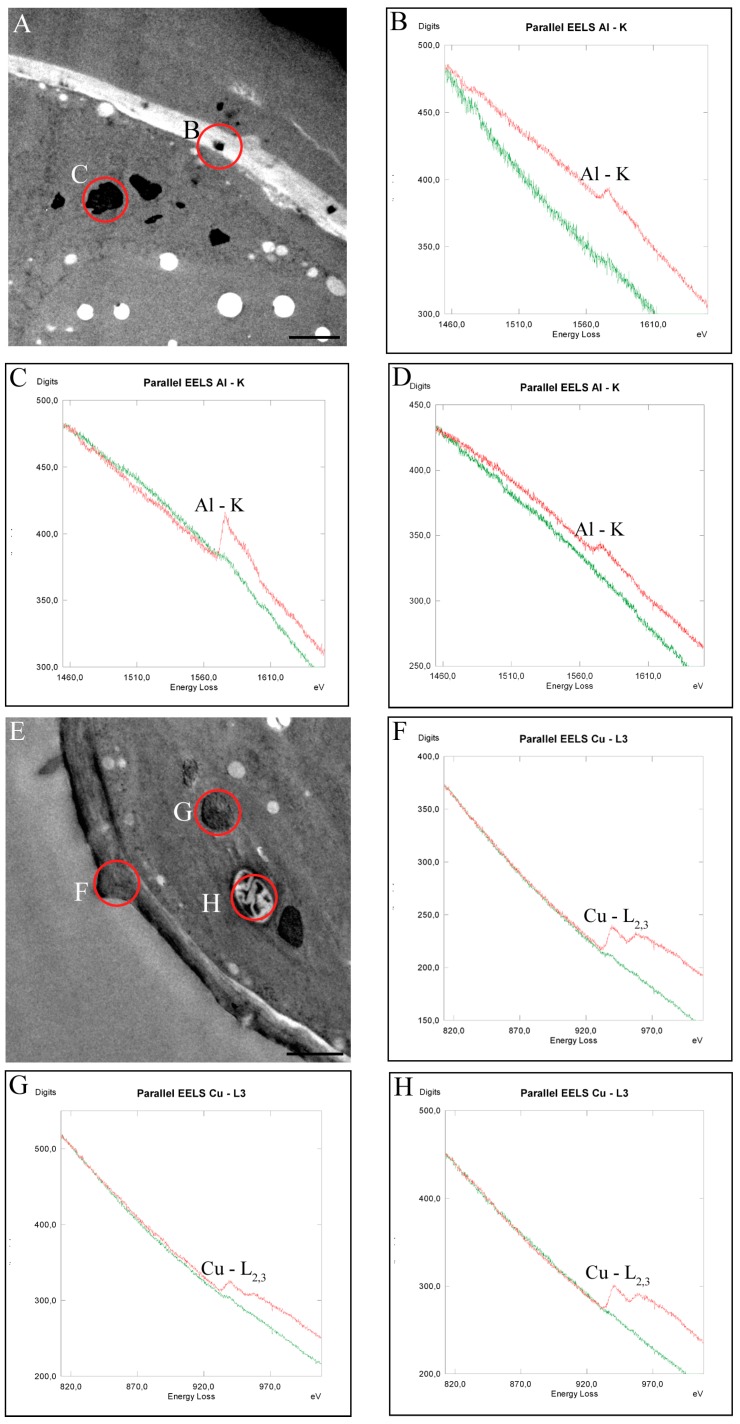

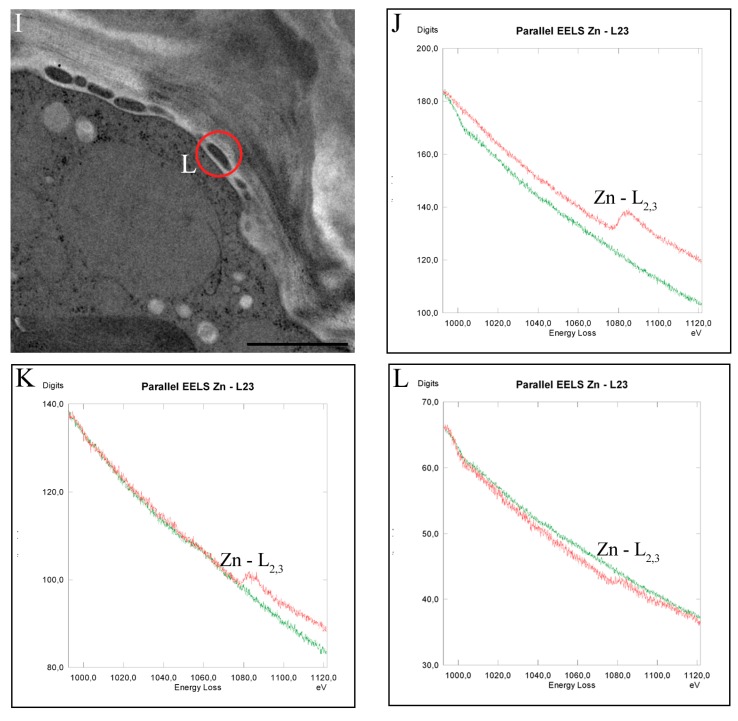
TEM micrographs of measurement sites (**A**,**E**,**I**) and EEL-spectra measured in *Desmidium* cells treated with 10 µM Al_2_(SO_4_)_3_ (**B**–**D**), with 0.3 µM CuSO_4_ (**F**–**H**), and with 10 µM ZnSO_4_ (**J**–**L**), respectively, for 21 days. Measurement areas of the single spectra are indicated as circles in the respective TEM micrographs. The red graphs represent the measurements indicated, the green graphs show control spectra; (**B**) Al is detected by the K-edge in a precipitation in the secondary cell wall; (**C**) in dark inclusions in the chloroplast and (**D**) in starch grains (measurement area not shown in TEM micrograph); (**F**) Cu is measured by L_2,3_-edge in the secondary cell wall; (**G**) in electron dense inclusion in the chloroplast and (**H**) in a starch grain; (**J**) Zn L_2,3_-edges were identified in the cell wall itself; (**K**) in electron dense inclusions of the chloroplast (measurement areas for (**J**,**K**) not shown); and (**L**) In abnormal depositions underneath the secondary cell wall. Bar = 2 µm.

In TEM, metal induced structural abnormalities could be detected in different organelles (Table 1). Al induced electron dense inclusions in the chloroplast (Figure 6B) that were not present in controls (Figure 6A). Additionally electron dense precipitations were found in the cell wall of Al-treated cells (Figure 7A). In Cd-exposed *Desmidium* filaments mitochondria showed an abnormal crystalline core possibly representing densely packed enzymes (Figure 6C). Additionally, autophagosome-like structures were present in the cytoplasm after Cd exposure (Figure 6D,E). The cytoplasm of CrVI-treated cells was not changed (Figure 6F) when compared to controls (Figure 6A). However, characteristic bag-like depositions with vesicular inclusions were found at irregular distances underneath the secondary wall (Figure 6G). Similar evaginations at the cytoplasmic side of the secondary wall were also present when *Desmidium* filaments were grown in Cu (Figure 6H) or Zn (Figure 6I). In the latter case these structures sometimes were increased to massive “spongy” cell wall accumulations (Figure 6I). Immunogold labeling showed that the wall accumulations after Zn treatment were not labeled by BG1, indicating that their composition differs from that of the secondary cell wall (Figure 3H).

In Cu-exposed *Desmidium* filaments abnormal vacuole-like structures (Figure 6K) and electron dense inclusions (Figure 7E) appeared in the chloroplast, which were not present in controls (Figure 6A).

Mitochondria of Zn-treated cells were up to four times longer than mitochondria of controls and their cristae were distinctly enlarged and bloated (Figure 6J; compare control in Figure 6A).

### 2.5. Intracellular Heavy Metal Localization by Electron Energy Loss Spectroscopy (EELS) and Electron Spectroscopic Imaging (ESI) in TEM

In order to obtain insight into intracellular metal distribution and sequestration and possible metal targets after 21 days treatment of *Desmidium* with the different metals, analyses by TEM-coupled EELS were performed. Al was detected in precipitations of the cell wall, in electron dense inclusions in chloroplast and in starch grains (Figure 7A–D, see also Table 1).

Cu was also found in the cell wall, in randomly distributed electron dense inclusions of the chloroplast, and additionally in starch grains (Figure 7E–H).

Distinct Zn sequestrations were measured after 21 days treatment in the cell wall itself, in dark inclusions in the chloroplast as well as in abnormal cell wall deposition (Figure 7I–L). CrVI could be measured in *Desmidium* only in trace amounts in cell wall evaginations by EELS. Cd could not be measured in any cell compartment of *Desmidium* or in the cytoplasm.

**Figure 8:**
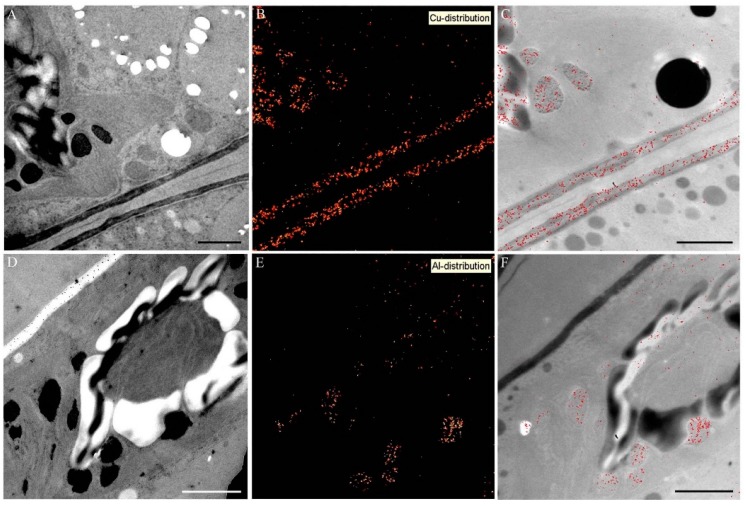
Element distribution visualized by electron spectroscopic imaging (ESI) in *Desmidium* filaments treated with 0.3 µM CuSO_4_ (**A**–**C**) and 10 µM Al_2_(SO_4_)_3_ (**D**–**F**) for 21 days; (**A**,**D**) TEM micrographs of *Desmidium* cells; (**B**,**E**) element distribution by ESI in red and (**C**,**F**) superimposition of TEM images and corresponding element distribution. Bar = 1 µm.

Element mapping by ESI confirmed the presence of Cu in cell walls and in electron dense inclusions, starch grains and in the chloroplast (Figure 8A–C) and of Al in the dark inclusions of the chloroplast (Figure 8D–F). Zn could not be detected at any site of the cell by means of ESI. This is probably due to the lower sensitivity of ESI in comparison to EELS, which does not allow detection of low element concentrations.

### 2.6. Determination of Al and Zn Distribution via Morin Fluorescence in CLSM

**Figure 9:**
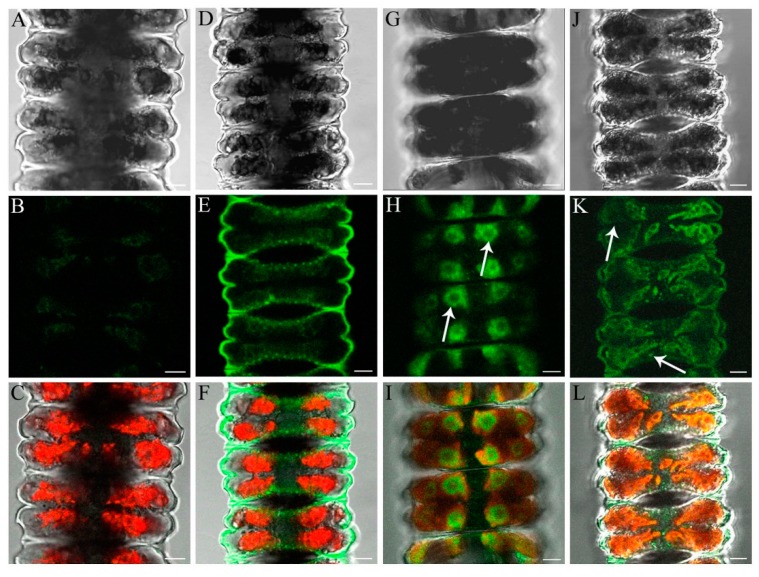
Detection of the distribution of Al and Zn in *Desmidium* filaments by morin fluorescence in CLSM, in untreated controls and after exposure of the algae to 50 µM Al_2_(SO_4_)_3_ as well as 10 and 50 µM ZnSO_4_, respectively. The first line shows digital interference contrast (DIC) images, the second line presents the morin labeling only, and the third line indicates the merged images (including chloroplast autofluorescence in red). (**A**–**C**) Controls show no morin staining; (**D**–**F**) *Desmidium* filaments treated with Al for 1 h show fluorescence of the cell wall; (**G**–**I**) After Zn exposure (50 µM) for 1.5 h, pyrenoid regions with surrounding starch grains are labeled (arrows); (**J**–**L**) After 21 days treatment with Zn (10 µM), the cell wall and a few spots near the chloroplast are stained (arrows). Bars = 5 µM.

Untreated control cells showed no labeling by morin (Figure 9A–C). Al was detected in the cell wall by morin fluorescence after short-term incubation (Figure 9D–F). Zn was only detected in chloroplast regions of pyrenoids (Figure 9G–I) during short-term treatment, and in the cell wall after long-term exposure where additionally fluorescent spots were found near the chloroplast (Figure 9J–L).

## 3. Discussion

Our results show that experimental exposure to the highest tolerated metal concentrations of Al, Cu and Zn severely harm the filamentous peat bog alga *Desmidium* in respect to biomass production, growth and ultrastructure. Moreover, it is demonstrated by means of analytical TEM (energy filtering) that all three metals are not only deposited in the cell wall but enter the cytoplasm and are sequestered in the chloroplast, yet to a different degree. Surprisingly, this intracellular uptake is reflected in an only slight decrease in photosynthetic activity in the case of Al and Cu, whereas Zn induced a marked drop in efficiency of PSII. These results also correspond to intracellular structural alterations in response to these three metals.

Whereas, Al and Cu only evoke ultrastructural changes at the cell wall and in the chloroplast, Zn additionally affected mitochondria and accordingly leads to a dieback of the algal filaments indicated by a distinctly negative value in biomass production within a period of three weeks. The severe impact of Cr and Cd on biomass production and ultrastructure in *Desmidium* goes along with the fact that both metals, though obviously readily taken up into the cells, are not or almost not detectable by our sensitive analytical TEM methods. By corroborating our earlier results in *Micrasterias* [3,4], this clearly points towards a link between the capability of a cell to compartmentalize a metal and the degree of its toxicity. Cd and Cr, which were not found to be compartmentalized in both algae, were most toxic [3,13]. Mitochondria and chloroplast structure were disturbed and as a consequence photosynthetic activity was markedly reduced particularly during Cd exposure. Correspondingly, Al and Cu, which were shown to be compartmentalized the best, both in *Micrasterias* [13] and *Desmidium* (this study), allowed almost normal photosynthetic activity and a still positive, though reduced, biomass production. By sequestering Al, Zn and Cu the cell wall seems to play an important role in metal detoxification in addition to intracellular compartmentalization. This has been also shown in other algae such as *Chara* [17], *Trebouxia* [18], *Thallassiosira* [19] and many others. Cell wall composition may affect the ability to accumulate metals [20] and metals may replace Ca from its natural bonding to pectins [13,21,22,23].

Comparison between the multicellular desmid *Desmidium* and the unicellular model system *Micrasterias* in their ability to cope with metal pollution shows both similarities and marked differences (Table 1). Generally, when comparing effects of the highest tolerable concentrations, Al and Cu were less toxic to both algae followed by Zn. Cr and Cd produced the most pronounced negative effects on biomass production or cell division rates, respectively and caused marked structural damage to organelles. Tolerance of *Desmidium* to the metals Al and Zn was distinctly lower than that of *Micrasterias*, whereas the metals Cu, Cd and CrVI were tolerated in both algae to a similar degree (Table 1). In contrast, interestingly, reduction of photosynthetic activity in CrVI-exposed *Desmidium* filaments was less distinct than expected from the other data (biomass production, ultrastructural alterations) and less pronounced than in *Micrasterias.* The relatively fair values of photosynthetic activity (*F*_v_/*F*_m_) for CrVI and Zn in comparison to the drastically reduced biomasses seem to indicate that the algae, though not able to divide any longer, are still in good physiological condition.

Both in *Micrasterias* [3] and in *Desmidium*, Cd was the only metal that induced autophagy probably indicating that the cells underwent programmed cell death. The severe disintegrating effects of Cd on dictyosomes in *Micrasterias* could not be confirmed in *Desmidium.* This is at least in part due to the fact that the few small Golgi bodies in *Desmidium* are inactive during interphase and are difficult to detect in TEM, whereas *Micrasterias* has a much greater number of large Golgi bodies producing large mucilage vesicles in all cell stages.

Differences in metal uptake between the two different algae were detected in the case of Al in TEM (Table 1). In *Micrasterias* Al was only measured in the cell wall by EELS, while in *Desmidium* Al is taken up into the cytoplasm and is sequestered in the chloroplast and in starch grains in addition to the cell wall. As the physiological and structural response of both algae to Al was similar, we suppose that the differences in metal localization in this instance are just a matter of concentration. The ultrastructural changes occurring as a consequence of Al exposure in *Micrasterias* (structural disintegration and morphological change in dictyosomes; [13]) indicate that low concentrations of Al must have been taken up into the cytoplasm as well but were probably not detectable by EELS. On the other hand, the fact that morin staining did not show any intracellular Al in *Desmidium* indicates that only low concentrations of Al are taken up into the cytoplasm that are below the detection limit of the fluorescent labeling but are detectable by TEM coupled EELS.

**Table 1:** Comparison of metal effects between the multicellular filamentous alga *Desmidium* and the unicellular alga *Micrasterias*. Data for *Desmidium* taken from this study, for *Micrasterias* from Volland *et al.* [4,13] and from Andosch *et al.* [3]. For details see Section 4.8., statistics are given in original publications. Chl: Chloroplast, cw: Cell wall, cwd: Cell wall depositions, d: Dictyosomes, sg: Starch grains, m: Mitochondria, mv: Mucilage vesicles, vac: Vacuole.

Metal	Object	Highest Tolerable Concentration, 21 Days	Cell Division/Biomass Production in %, 21 Days	Ultrastructural Changes	Intracellular Localization Determined by EELS	Photosynthetic Electron Transport (PSII) *F*_v_/*F*_m_ in %, 21 Days
control	*Desmidium*	0	100			100
	*Micrasterias*	0	100			100
Al	*Desmidium*	10 µM	40	dark precipitations in chl and cw	chl, sg, cw	91
	*Micrasterias*	20 µM	28	d involute and partly disintegrated, increased vacuolization	cw	97
Cu	*Desmidium*	0.3 µM	69	bag-like cwd, dark precipitations and vac in chl	chl, sg, cw	89
	*Micrasterias*	0.3 µM	56	precipitations in sg and cw, increased vacuolization	sg, cw, mv	102
Cd	*Desmidium*	0.6 µM	27	m with crystalline core autophagosomes	not detectable	33
	*Micrasterias*	0.6 µM	15	d morphologically severely changed and disintegrated, autophagosomes	not detectable	26
CrVI	*Desmidium*	10 µM	−23	bag-like cwd	traces	84
	*Micrasterias*	10 µM	18	bag-like cwd, increased vacuolization, disturbed chl structure	cw-bags	41
Zn	*Desmidium*	10 µM	−90	dark precipitations in chl, bag-like cwd, enlarged and bloated m	chl, cw, cwd	61
	*Micrasterias*	30 µM	0	dark precipitations in cw, electron dense vac, distrubed chl structure	vac, mv, cwd	76

TEM analyses of different cell growth stages of *Desmidium* revealed differences in morphogenesis when compared to well-known desmids such as *Micrasterias*. These differences, which mainly concern cell wall formation, may account for the different behavior in metal uptake and sequestration. Whereas in *Micrasterias* the cell shape is modulated by the primary wall, which later is underlaid by a rigid secondary wall, the secondary wall participates in morphogenesis of *Desmidium* cells. Similar cell shaping modes have been also reported in another species, *Desmidium* *baileyi* [15] as well as in earlier studies on the filamentous desmids *Bambusia* [24,25] or *Onychonema* [26]. The final shape in *Desmidium* results from semicell expansion (splitting of the connecting primary wall, unfolding of the cylinder and rounding at the edges, see Figure 1 and Figure 2), after secondary wall formation. At least at the time of semicell expansion, the secondary wall of *Desmidium* has to be rather elastic. This elasticity could arise from different texture, from the fact that the secondary wall is not yet fully developed or from different chemical composition. At least in respect to hemicelluloses ((1-3, 1-4)-ß-d-glucans), the latter does not seem to be the case as our labeling with BG1 antibody in *Desmidium* did not indicate differences to the secondary wall of *Micrasterias* (see [21,27]). Nevertheless, the higher flexibility of the cell wall in *Desmidium* may possibly cause higher penetrability and thus less effective filter properties of the cell wall for metals. This would explain why Al is taken up into the cytoplasm of *Desmidium* more readily than into that of *Micrasterias*.

We conclude from our study that Al, Cu, Cd, Cr and Zn extremely reduce biomass production and viability of the filamentous bog alga *Desmidium*, with Cd and CrVI evoking the most toxic effects. All metals investigated are taken up into *Desmidium* cells, are accumulated in cell walls and in the cytoplasm, and cause severe effects on ultrastructure. Comparison of the highest tolerable metal concentrations in long-term experiments (three weeks) clearly showed that the filamentous, multicellular *Desmidium* is considerably more sensitive to the metals Al and Zn than the unicellular *Micrasterias*. As *Desmidium* contributes to biomass production in a bog much more than *Micrasterias*, this means that Al and Zn in addition to the highly toxic Cd and CrVI may pose severe threats to the ecosystem.

## 4. Experimental Section

All chemicals are purchased from Sigma-Aldrich (Vienna, Austria) or Roth (Karlsruhe, Germany) unless stated otherwise.

### 4.1. Cell Cultures

The filamentous alga *Desmidium swartzii* was grown under semi-sterile conditions in a liquid Desmidiaceaen medium [28] or on agar plates with an agar concentration of 0.5%. For liquid cultivation, *Desmidium* filaments were inoculated into 30 mL culture medium in 100 mL Erlenmeyer flasks. Cultures were kept at constant temperature of 20 °C, a 14/10 h light-dark regime and a light intensity of approx. 50 µmol·photons·m^−2^·s^−l^. Philips 58W/827 tubes were used for illumination. For all experiments, cells were subcultured every 3–5 weeks and were used about 3 weeks after subculturing unless stated differently.

### 4.2. Heavy Metal Treatments and Determination of Highest Tolerable Metal Concentration

In order to determine the highest tolerated metal concentration for the subsequent experiments, metal concentrations used for the unicellular desmid *Micrasterias* in earlier studies [4,13] were tested and modified for *Desmidium.* For short- and long-term exposures concentrations and time periods were taken that evoke distinct light microscopically visible effects without leading to necrosis. For short-term treatment (1–2 h), 50 µM Al_2_(SO_4_)_3_ and 50 µM ZnSO_4_ were used (see Section 4.7.). For long-term application (3 weeks), 10 µM Al_2_(SO_4_)_3_, 0.6 µM CdSO_4_, 10 µM K_2_Cr_2_O_7_ (CrVI), 0.3 µM CuSO_4_, 10 µM ZnSO_4_, respectively, as well as 10 µM CrVI (5 weeks), were applied. *Desmidium* filaments were kept in the same metal solution during the entire experiment.

### 4.3. Biomass Production

Al, Cd, Cr, Cu and Zn were added to the liquid culture medium in 100 mL Erlenmeyer flasks containing a defined amount of *Desmidium* filaments (0.7 g fresh weight each) to obtain final concentrations of 10 µM Al_2_(SO_4_)_3_, 0.6 µM CdSO_4_, 10 µM CrVI, 0.3 µM CuSO_4_ respectively 10 µM ZnSO_4_. The samples were exposed to constant culture conditions (see Section 4.1.). The same amount of untreated control algae was grown in pure culture medium under the same conditions. After 21 days fresh weight was determined from untreated controls and treated samples. The experiment was repeated in triplicate. Standard errors are given.

### 4.4. Light Microscopy

For light microscopic analysis of cell growth and morphogenesis, controls and algae treated with different metal concentrations for varying time periods (see Section 4.2.) were investigated in a light microscope (UNIVAR, Reichert, Vienna, Austria) and photographed with a Canon Power Shot G5 camera (Canon, Vienna, Austria). To improve the images CombineZM image stacking Software (Alan Hadley, UK) was employed.

### 4.5. Determination of Photosynthetic Electron Transport Efficiency (PSII)

Photosynthetic electron transport efficiency was measured in *Desmidium* cultures exposed to 10 µM Al_2_(SO_4_)_3_, 0.6 µM CdSO_4_, 10 µM CrVI, 0.3 µM CuSO_4_ or 10 µM ZnSO_4_ for 3 weeks and in controls by recording fast chlorophyll fluorescence [29] with a Handy Pea (Hansatech, King’s Lynn, UK). A minimum of 6 assays was measured per treatment and control. For methodological details see [30,31].

### 4.6. TEM Analysis, EELS and ESI Measurements and Immuno TEM

For electron microscopy, untreated *Desmidium* filaments and algae treated with 10 µM Al_2_(SO_4_)_3_, 0.6 µM CdSO_4_, 10 µM CrVI (35 days ), 0.3 µM CuSO_4_ or 10 µM ZnSO_4_ for 21 days were cryofixed in a Leica EMPACT high pressure freezer, freeze-substituted in a Leica EM AFS (Leica Microsystems, Vienna, Austria) as described earlier [32,33] and embedded in Agar low viscosity resin (LV Resin, VH1 and VH2 Hardener and LV Accelerator; Agar Scientific, Essex, UK) or LR-White (London Resin Company Ltd., Theale, UK). Probes were sectioned on a Reichert Ultracut (Reichert, Vienna, Austria) or a Leica UC7 (Leica Microsystems, Vienna, Austria). For conventional imaging 80 nm sections were placed on Formvar coated copper grids or on gilded copper grids for immuno-TEM. For EELS and ESI 40 to 50 nm thick sections were mounted on hexagonal narrow mesh uncoated copper grids. Sections were investigated in a LEO 912 AB TEM (Zeiss, Oberkochen, Germany) with in-column energy filter at 80 kV for conventional imaging (zero-loss energy filtering) and immuno-TEM, and at 120 kV for EELS and ESI. Images were acquired by a TRS Sharpeye dual speed slow scan CCD camera (Tröndle, Mohrenwies, Germany) run by ITEM Software (SIS, Soft Imaging System, Münster, Germany). The magnification for EELS was between ×25,000 and ×63,000, illumination angles between 1.6 and 4 mrad and camera exposure time between 1 and 5 s (with 7 integration cycles). The measured area for EELS was defined by using a 100 µm spectrometer entrance aperture with a spectrometer magnification of ×163 for Zn-treated cells and ×125 for the other measurements. Al was identified by the K-edge at 1577 eV, Cu by the L_2,3_-edge at 935 eV, Zn by the L_2,3_-edge at 1073 eV, Cd by M_4,5_-edge at 424 eV and Cr by L_2,3_-edge at 570 eV. Elemental maps for ESI were calculated using the three windows power-law method; see also [34].

For immunogold labeling of cell wall constituents the following monoclonal antibodies were used: JIM 8 and JIM13 (PlantProbes, Leeds, UK) against epitopes of arabinogalactans, JIM5 and JIM7 (PlantProbes, Leeds, UK) against low and highly methyl-esterified pectins, BG1 (Biosupplies, Parkville, Australia) against (1-3, 1-4)-ß-d-glucans. Sections were blocked in Trizma-buffer (pH 7.4) containing 1% BSA (bovine serum albumin) and 0.1% Tween 20 (polyoxyethylene-sorbitan monolaurate). Incubation in the primary antibody overnight at 4 °C (Jim5 and Jim7 undiluted, JIM13 diluted 1:20 and BG1 1:80) was followed by four washing steps and incubation in the secondary antibody (anti-rat IgG, 1:40 for JIM antibodies and for BG1 anti-mouse IgG 1:40, both conjugated with 10 nm gold) at room temperature for 1 h. Finally the grids were washed twice and rinsed with buffer, with distilled water and were then dried.

### 4.7. Detection of Al and Zn by Morin Fluorescence in CLSM

In order to complement the results from analytical TEM, morin hydrate, a fluorochrome that forms fluorescent complexes with Al and Zn was used to analyze Al and Zn distribution in filaments of *Desmidium.*

Algae were exposed to 50 µM Al_2_(SO_4_)_3_ × 16 H_2_O and 50 µM ZnSO_4_ for short-term treatment (1, 1.5 h) and to 10 µM ZnSO_4_ for long-term experiments (21 days) in their culture medium. Treated algae and controls were washed twice and then stained with 100 µM morin hydrate for 15 min. After washing algal filaments were observed in a confocal laser scanning microscope (Zeiss LSM 510, Axiovert 100 M, Carl Zeiss, Jena, Germany) with an excitation of 488 nm (argon laser) and emission wavelengths between 505 and 550 nm band-pass filtered.

### 4.8. Comparison of Data between Desmidium and Micrasterias

In order to evaluate metal effects between the multicellular filamentous alga *Desmidium* and the unicellular alga *Micrasterias*, highest tolerable metal concentrations, cell division rates/biomass production, ultrastructural changes, intracellular metal concentrations as determined by EELS and photosynthetic electron transport efficiency were compared. Data on *Micrasterias* were taken from Volland *et al.* [4,13] and Andosch *et al.* [3], data on *Desmidium* from this study. To calculate cell division rates in *Micrasterias* for comparison to biomass production in *Desmidium*, the number of control cells was set to 100%. Values were taken from Figure 2A [13] and Figure 2B [4]. For comparison of efficiency of electron transport in PSII, *F*_v_/*F*_m_ values of controls were set to 100% both in *Micrasterias* (Table 1, [13] and Figure 3B, [4]) and in *Desmidium* (this study). For statistics see original publications.
