# Correction: Subcellular Sequestration and Impact of Heavy Metals on the Ultrastructure and Physiology of the Multicellular Freshwater Alga *Desmidium swartzii*

**DOI:** 10.3390/ijms160920239

**Published:** 2015-08-26

**Authors:** Ancuela Andosch, Margit Höftberger, Cornelius Lütz, Ursula Lütz-Meindl

**Affiliations:** 1 Plant Physiology Division, Cell Biology Department, University of Salzburg, Hellbrunnerstrasse 34, 5020 Salzburg, Austria; E-Mails: ancuela.andosch@sbg.ac.at (A.A.); margit.hoeftberger@sbg.ac.at (M.H.); 2 Institute of Botany, Faculty of Biology, University of Innsbruck, Sternwartestrasse 15, 6020 Innsbruck, Austria; E-Mail: cornelius.luetz@uibk.ac.at

In this recently published paper [1], the wrong Austrian Science Fund Project Number (210316-B16) was quoted in the Acknowledgment Section. The correct version of the Acknowledgment is given below.
